# Pathological regression patterns following neoadjuvant chemo-immunotherapy in head and neck squamous cell carcinoma: a pilot study

**DOI:** 10.3389/fimmu.2025.1627442

**Published:** 2025-08-06

**Authors:** Dikan Wang, Fanning Zeng, Sien Zhang, Wanming Hu, Yahui Wang, Daiqiao Ouyang, Bin Zeng, Guozhong Zeng, Jingyuan Li, Guiqing Liao, Yujie Liang

**Affiliations:** ^1^ Hospital of Stomatology, Guanghua School of Stomatology, Sun Yat-sen University, Guangzhou, Guangdong, China; ^2^ Guangdong Provincial Key Laboratory of Stomatology, Sun Yat-sen University, Guangzhou, Guangdong, China; ^3^ Department of Pathology, State Key Laboratory of Oncology in South China, Guangdong Provincial Clinical Research Center for Cancer, Sun Yat-Sen University Cancer Center, Guangzhou, Guangdong, China

**Keywords:** chemo-immunotherapy, neoadjuvant therapy, head and neck squamous cell carcinoma (HNSCC), regression pattern, pathological response

## Abstract

**Introduction:**

Neoadjuvant chemoimmunotherapy (NACI) has drawn considerable attention in Head and neck squamous cell carcinoma (HNSCC) owing to its potential in functional preservation and treatment-failure reduction. Yet whether the surgical extent can be narrowed following NACI is largely debatable due to a potential non-centripetal tumor regression may result in scattered microfoci residing beyond the narrowed margin.

**Methods:**

In this pilot study, we characterized the tumor regression pattern in a post-NACI HNSCC cohort using a whole-mount histopathological approach. The MRI examinations before and after NACI were used to evaluate the objective response rate (ORR).

**Results:**

Of the 52 patients enrolled, the ORR was 75%. Pathological complete response (pCR) rate was 15.4%, and the major pathological response (MPR) rate was 40.4%. Two major regression patterns were identified in whole-mount tumor sections, centripetal regression and non-centripetal regression. Centripetal regression was observed in 37 patients (71.2%) and was subcategorized into complete regression (Ia, 15.4%), unifocal centripetal regression (Ib, 36.5%), and multifocal centripetal regression (Ic, 19.2%). Non-centripetal regression was seen in 15 patients (28.8%) and was subcategorized into scattered regression (IIa, 25.0%) and non-regression (IIb, 3.8%). Moreover, we found a pre-NACI CPS higher than 20 or post-NACI (18)F-FDG SUVmax reduction exceeding 50% were potential predictive factors for the centripetal regression pattern.

**Discussion:**

We revealed for that centripetal regression was the predominant pattern of regression after NACI in HNSCC. Hence, our data presumably supports a reduced surgical extent in post-NACI HNSCC patients. Future studies should focus on identifying accurate predictive factors for the regression pattern, which may eventually assist in risk stratification and surgical decision making.

**Conclusions:**

The pattern of tumor pathological regression after NACI for HNSCC is mainly divided into centripetal and non-centripetal regression, with the former accounting for the major portion of the regression.

## 1 Introduction

Head and neck cancer is one of the most prevalent cancers worldwide, with an estimated number of over 940,000 new cases and 480,000 deaths in 2022 (1). Head and neck squamous cell carcinoma (HNSCC) arises from the mucosal epithelium of the oral cavity, pharynx, and larynx and is the most prevalent cancer type in the head and neck region (2). Recent data from KEYNOTE-048 supported the use of pembrolizumab with or without chemotherapy for first-line treatment of recurrent/metastatic (R/M) head and neck squamous cell carcinoma (3, 4). Since then, anti-PD-1 immunotherapy has emerged as a rapidly evolving field in HNSCC, with diverse treatment modalities being proposed and tested in clinical trials (5). Nevertheless, surgery-centered multi-disciplinary treatment remains the cornerstone of management for locally advanced HNSCC (6). Head and neck region is anatomically highly complex and serves the primary vital and social functions (such as eating, speaking, and breathing), radical surgery (even with concurrent flap reconstruction) often results in large tissue and organ defects, which further lead to serious functional impairment and compromised quality of life. Therefore, applying immunotherapy in the neoadjuvant setting of HNSCC have drawn considerable attention, owing to its potential benefits in organ and functional preservation, local and distant failure reduction, and treatment de-escalation (7–10). Among various treatment modalities, neoadjuvant chemoimmunotherapy (NACI) is deemed to be a promising choice. NACI have been shown to achieve superior surgical, pathological, and efficacy outcomes compared to traditional neoadjuvant chemotherapy in solid tumors (11, 12). In addition, multiple clinical trials have reported a high objective response rate (ORR) and major pathological response (MPR) after NACI in HNSCC (8, 13–15).

However, whether the extent of surgical resection can be reduced following NACI is largely debatable. The primary concern is that the tumor may regress in a non-centripetal fashion, potentially leaving behind scattered microfoci residing away from the tumor epicenter. Under such circumstances, if a reduced surgical margin is implemented, these microfoci may persist beyond the surgical margin, ultimately resulting in recurrence or metastasis (5, 16). Multifocal tumor regression following neoadjuvant chemotherapy has been previously described in breast, oral, esophagus and colorectal cancer (16–20). Moreover, a multifocal tumor-regression pattern has been associated with locoregional recurrence in breast and oral cancer (16, 21). Yet in the era of immunotherapy, with the ORR being dramatically improved, the tumor-regression pattern following NACI might be largely different (19).

To date, the pathological regression patterns of HNSCC following NACI have not been systematically characterized. Therefore, the primary objective of this study was to characterize and categorize these regression patterns using a whole-mount histopathology approach. Additionally, we tried to identify the potential predictive factors for centripetal regression after NACI in HNSCC.

## 2 Methods and materials

### 2.1 Study design and patients

This study was approved by the Ethics Committee of Stomatological Hospital of Sun Yat-sen University (Approval Number: KQEC-2023-94-01). A total of 52 patients with head and neck squamous cell carcinoma (HNSCC) who received neoadjuvant immunochemotherapy followed by surgery at the Stomatological Hospital of Sun Yat-sen University from May 2023 to May 2024 were included in the current study. Informed consents were obtained from all participants. This study was performed in accordance with the 1964 Helsinki Declaration (along with its later amendments or similar ethical standards).

Eligible patients were aged between 18–80 years, with histologically confirmed as locally advanced (stage III-IVb according to the 8th Edition of American Joint Committee on Cancer [AJCC] guideline) primary or recurrent/metastatic HNSCC. Other inclusion criteria included: at least one measurable lesion according to the modified Response Evaluation Criteria in Solid Tumors (RECIST) version 1.1 for immune based therapeutics (iRECIST), and adequate organ function. Patients were excluded if they had severe comorbidities (including severe cardiac insufficiency, renal or hepatic dysfunction, uncontrolled diabetes, autoimmune disease, and mental disorders), surgical contraindications, currently using immunosuppressive medication, or had a history of antitumor therapy. Infectious disease did not prevent the patients from enrollment except for HIV infection. The HPV status was determined by immunohistochemical staining of p16. All patients were evaluated by a head and neck surgeon before enrollment.

### 2.2 Treatment procedure and response assessment

Enrolled patients received NACI with 200mg pembrolizumab or tislelizumab plus albumin-bound paclitaxel (260 mg/m^2^) and cisplatin (75 mg/m^2^) on day one of each three-week cycle. All patients received two cycles of NACI following by radical surgery at our institution, except for one patient with implanted heart stent who received three cycles of NACI before surgery. Treatment was discontinued in the occurrence of unacceptable toxicity or disease progression. Symptomatic treatment was provided for adverse effects during the treatment process. Two to three weeks after completing pre-operative NACI, patients received comprehensive assessments, including MRI, followed by surgical resection of the tumor and cervical lymph node dissection. According to the National Comprehensive Cancer Network (NCCN) Clinical Practice Guidelines, some patients also received post-operative chemoimmunotherapy and/or radiotherapy.

According to the iRECIST standard, MRI-based radiological assessments were performed at baseline, after two cycles of treatment, and every six months thereafter. Based on iRECIST, tumor response was categorized into five types: iCR (complete disappearance of both target and non-target lesions); iPR (reduction of target lesions by ≥30%, with all lesions not meeting iCR or iUPD criteria); iSD (the criteria of iCR or iPR are not met and no tumor progression is present); iUPD (corresponds to sum of the tumor lesions increase by ≥20% compared with the nadir, non-target lesions progress and new lesions occur, which is not confirmed at the next imaging session within 4 to 8 weeks); iCPD (precisely determined by the following conditions at the 4 to 8 weeks imaging follow-up after iUPD, increase in target or non-target lesions by ≥5 mm or the appearance of new lesions) (22).

Throughout the study, adverse reactions were monitored and recorded, continued for 30 days after the last treatment. All adverse events were graded according to the Common Terminology Criteria for Adverse Events (CTCAE) version 5.0.

### 2.3 Whole-mount histopathology

Specimens of the primary tumor were obtained during surgery. We first analyzed the resected bulk tumor tissue and then incised along the maximum longevity axis of the center of the tumor (**Supplementary Figure S1**). The whole-mount specimen was taken by 2-3mm thick along the maximum length diameter of the tumor. Followed by fixation in paraformaldehyde for at least 24 h, fitting in cassettes, embedding in paraffin, and cutting into 4 μm thick whole-mount pathology slides. Immunohistochemical staining and hematoxylin-eosin staining were performed in whole-mount pathology slides of all post-treatment patients. Tumor cells were identified by anti-human Pan-Keratin (Pan-CK, CST #4545). Pretreated specimens were sequentially incubated with primary antibody (Pan-CK 1:200 dilution) and secondary antibody conjugated with horseradish peroxidase (HRP). The staining was visualized using Horseradish Peroxidase Color Development Kit (DAB), followed by hematoxylin staining the nuclei. The images of stained whole-mount pathology slides were acquired by the TissueFAXS platform (TissueGnostics, Austria) and analyzed by StrataQuest V7.0.1.140 (TissueGnostics, Austria). The images were identified of the presence of residual viable tumor cells within the tumor bed, to assist in the pathological assessment of tumor regression patterns, and to determine the pathological response. For the tumor pathologic response evaluation, it was classified as pathologic complete response (pCR), major pathologic response (MPR), partial pathologic response (PPR) and no pathologic response (NPR). PCR was defined as complete disappearance of viable tumor cells within the whole slide view; MPR was defined as <10% of residual viable tumor cells; PPR was defined as residual viable tumor cells between 10% and 80%; NPR was defined as >80% residual viable tumor cells (8).

### 2.4 Statistical analysis

Statistical analyses were performed using GraphPad Prism 8.0 and SPSS 27 software. Paired student’s t-test (two-tailed) was used to compare the pre- and post-therapeutic PET-CT SUVmax. Univariate logistic analysis was used to analyze the correlation between the tumor regression pattern and patients’ characteristics. The correlation between the SUVmax decrease rate and tumor regression pattern was analyzed by Chi-square test. Survival analysis was performed and presented by using the Kaplan–Meier method. The difference in survival curves was tested by the log-rank test. Quantitative data were showed as mean ± SEM unless stated otherwise. *P* < 0.05 was considered statistically significant.

## 3 Results

### 3.1 Patient’s characteristics

A total of fifty-two HNSCC patients were enrolled in this study. Demographics and clinicopathological characteristics are summarized in **Table 1**. Among them, 41 presented primary lesions, while 11 had recurrent/metastatic HNSCC. The median age was 55 (range: 32–75 years). Most patients were male (76.9%) and had tumors in the oral cavity (82.7). Eight patients were HPV positive. The clinical stage was frequently cT4 (61.5%) and cN+ (63.5%). Only three patients (5.8%) had a CPS score below 1, twenty-eight patients (53.8%) had a CPS score below 20, and twenty-one patients (40.4%) had a CPS score of 20 or higher. The follow-up duration ranged from 81 to 455 days, with a median follow-up time of 216 days.

**Table 1:** Patients’ demographic and clinical characteristics.

Characteristic	Patients (N=52)
**Age, median(range), years**	55 (32-75)
Gender, n (%)	
**Male**	40 (76.9)
**Female**	12 (23.1)
Primary/Recurrent/Metastatic, n (%)	
**Primary**	41 (78.8)
**Recurrent**	7 (13.5)
**Metastatic**	4 (7.7)
Smoking, n (%)	
**Yes**	31 (59.6)
**No**	21 (40.4)
Alcohol, n (%)	
**Yes**	24 (46.2)
**No**	28 (53.8)
Betel nut, n (%)	
**Yes**	11 (21.2)
**No**	41 (78.8)
Tumor subsite, n (%)	
**Tongue**	18 (34.6)
**Buccal**	10 (19.2)
**Gingival**	11 (21.2)
**Oropharyngeal**	5 (9.6)
**Floor of mouth**	3 (5.8)
**Palatal**	1 (1.9)
**Metastatic**	4 (7.7)
Clinical T stage, n (%)	
**T2**	1 (1.9)
**T3**	15 (28.8)
**T4**	32 (61.5)
**N/A (Metastatic)**	4 (7.7)
Clinical N stage, n (%)	
**N0**	15 (28.8)
**N1**	12 (23.1)
**N2**	16 (30.8)
**N3**	5 (9.6)
**N/A (Metastatic)**	4 (7.7)
Clinical TNM stage, n (%)	
**III**	12 (23.1)
**IV**	36 (69.2)
**N/A (Metastatic)**	4 (7.7)
HPV status	
**Positive**	8 (15.4)
**Negative**	34 (65.4)
**N/A**	10 (19.2)
PD-L1 combined positive score (CPS), n (%)	
**<1**	3 (5.8)
**1-20**	28 (53.8)
**≥20**	21 (40.4)

### 3.2 Safety and treatment response

Treatment related adverse events (TRAEs) were summarized in **Supplementary Table S1**. The top five TRAEs of any grade were alopecia (100%), fatigue (46.2%), rash (46.2%), hepatic dysfunction (42.3%) and anorexia (36.5%), and TRAEs of Grade 3 or worse included rash (3 cases), hepatic dysfunction (5 cases), myelosuppression (2 cases), and cardiotoxicity (1 case). No previous unknown or unexpected TRAEs were observed. All patients recovered following appropriate treatments, and no long-term adverse effects were observed. No TRAEs resulted in the discontinuation or dose reduction of studied drugs, or death.

**Figure 1:**
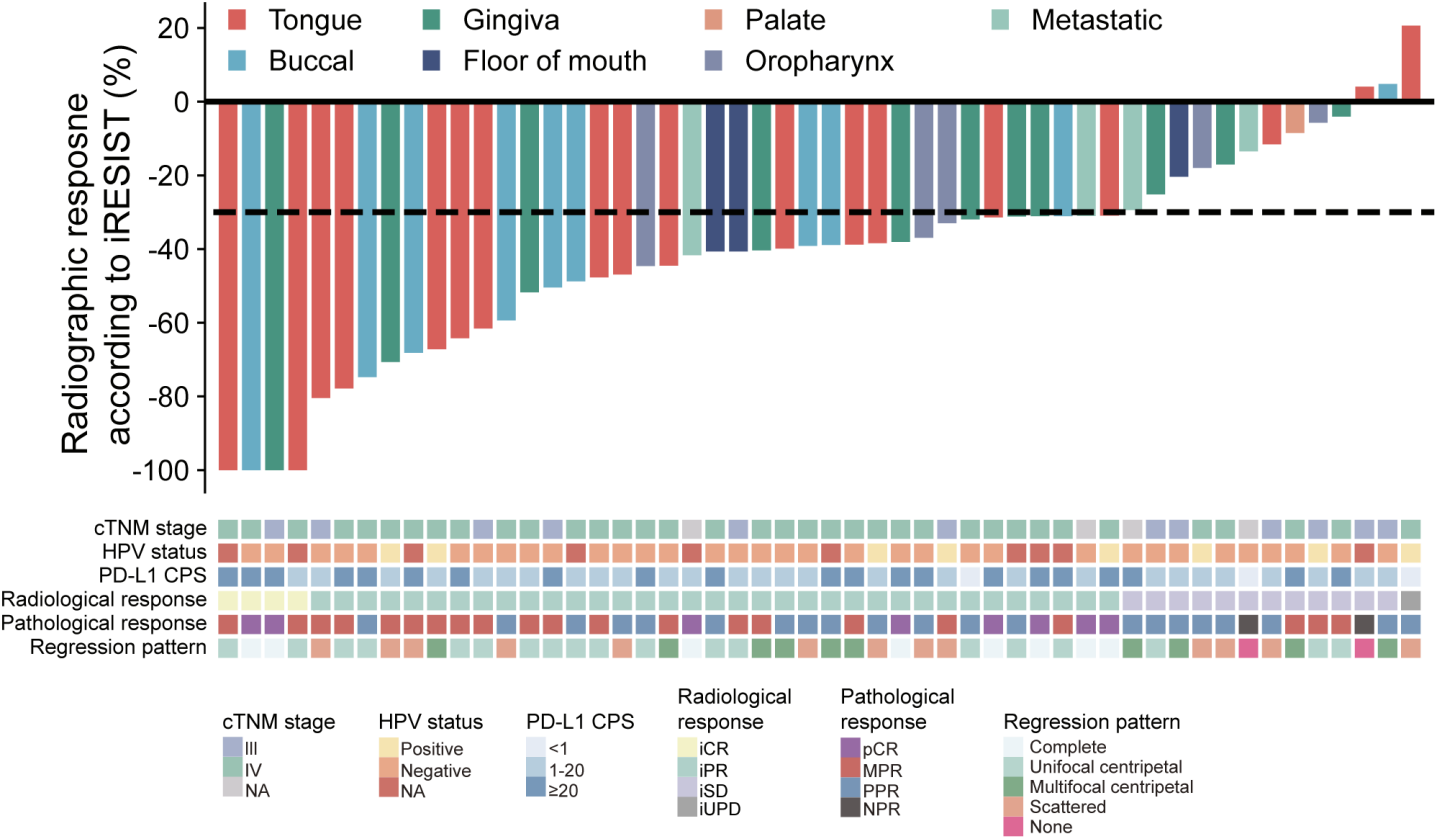
Waterfall plot of patients’ radiological response, pathological response, cTNM staging, HPV status, CPS score and regression pattern (n=52). Each bar indicates one patient.

According to the iRECIST criteria, among the 52 patients enrolled, 4 patients achieved an iCR (7.7%) and 35 patients (67.3%) achieved an iPR (**Figure 1**). The ORR was 75.0%. 12 patients (23.1%) had an iSD and one patient (1.9%) had an iUPD (**Figure 1**). No case with hyperprogressive disease was observed. Analysis of HE-stained whole-mounted tumor sections revealed pathological complete response (pCR) in 8 patients (15.4%), MPR in 21 patients (40.4%), partial pathological response (PPR) in 21 patients (40.4%), and no pathological response (NPR) in 2 patients (3.8%) (**Figure 1**). All patients received surgical treatment with a R0 resection after NACI. During follow-up, one patient died from distant metastasis, another patient died from uncertain reasons. The one-year overall survival rate was 95.8%. Two patients had local recurrence and lymphatic metastasis, respectively. The one-year disease-free survival was 90.1%. The remaining patients were alive without evidence of locoregional recurrence or lymphatic/distant metastasis.

### 3.3 Tumor regression pattern after NACI in HNSCC

**Figure 2:**
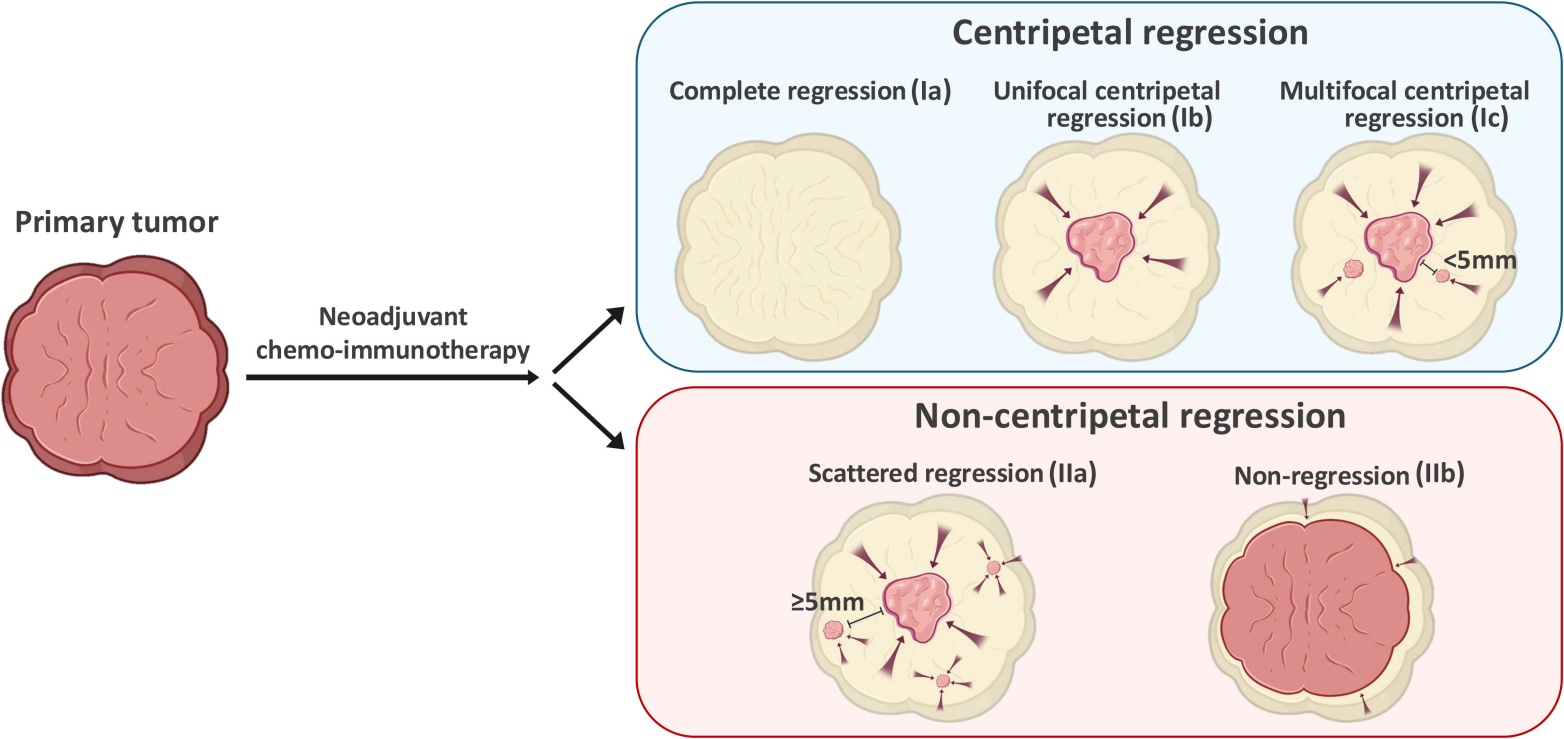
Schematic illustration of tumor regression patterns following NACI in HNSCC.

**Figure 3:**
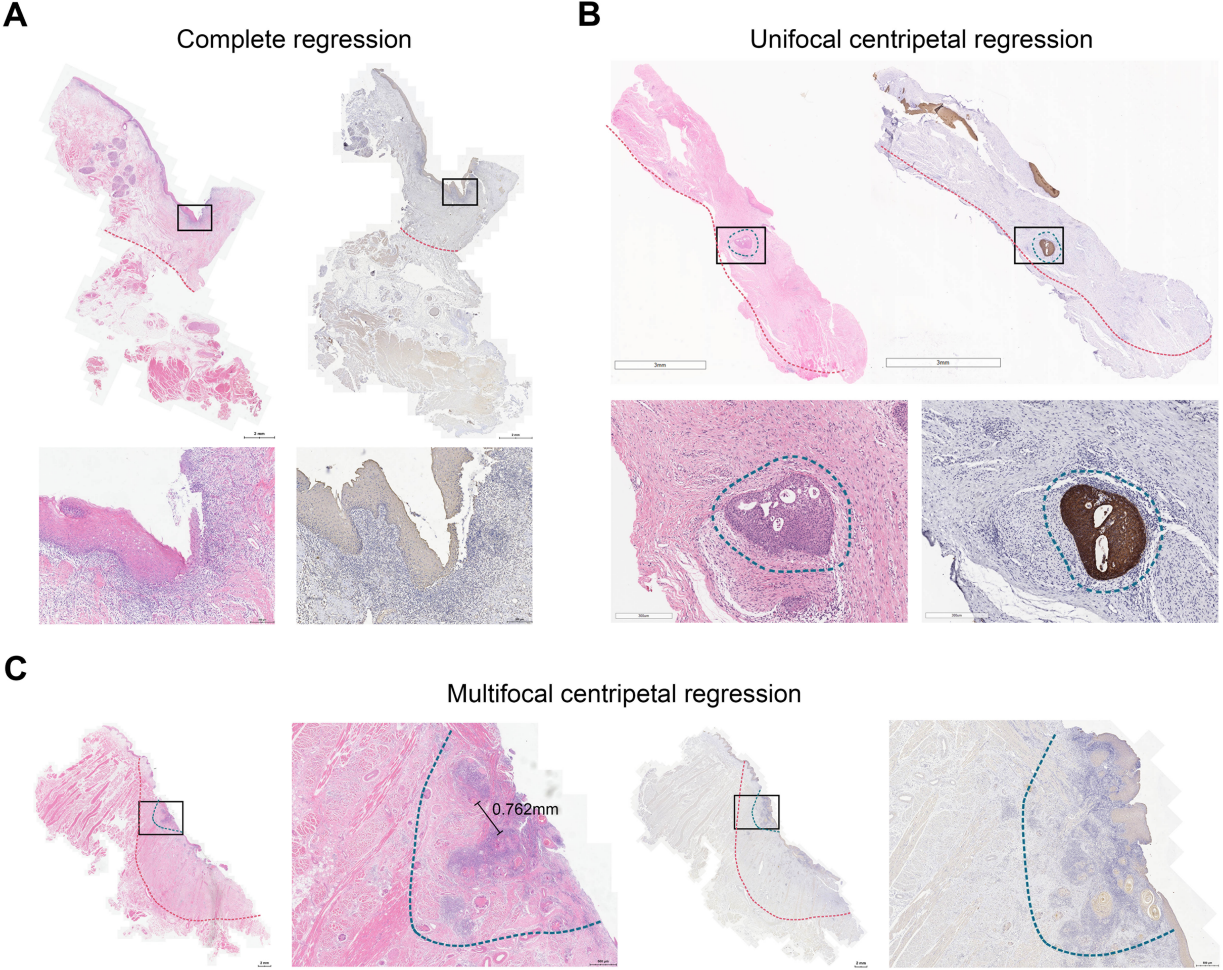
Centripetal regression following NACI. Representative image of whole-mount sections showing **(A)** complete regression (Ia), **(B)** unifocal centripetal regression (Ib), and **(C)** multifocal centripetal regression (Ic). Left panel: HE staining, right panel: pan-CK staining. The original tumor bed was outlined by red dotted line. The residual lesion was outlined by blue dotted line.

**Figure 4:**
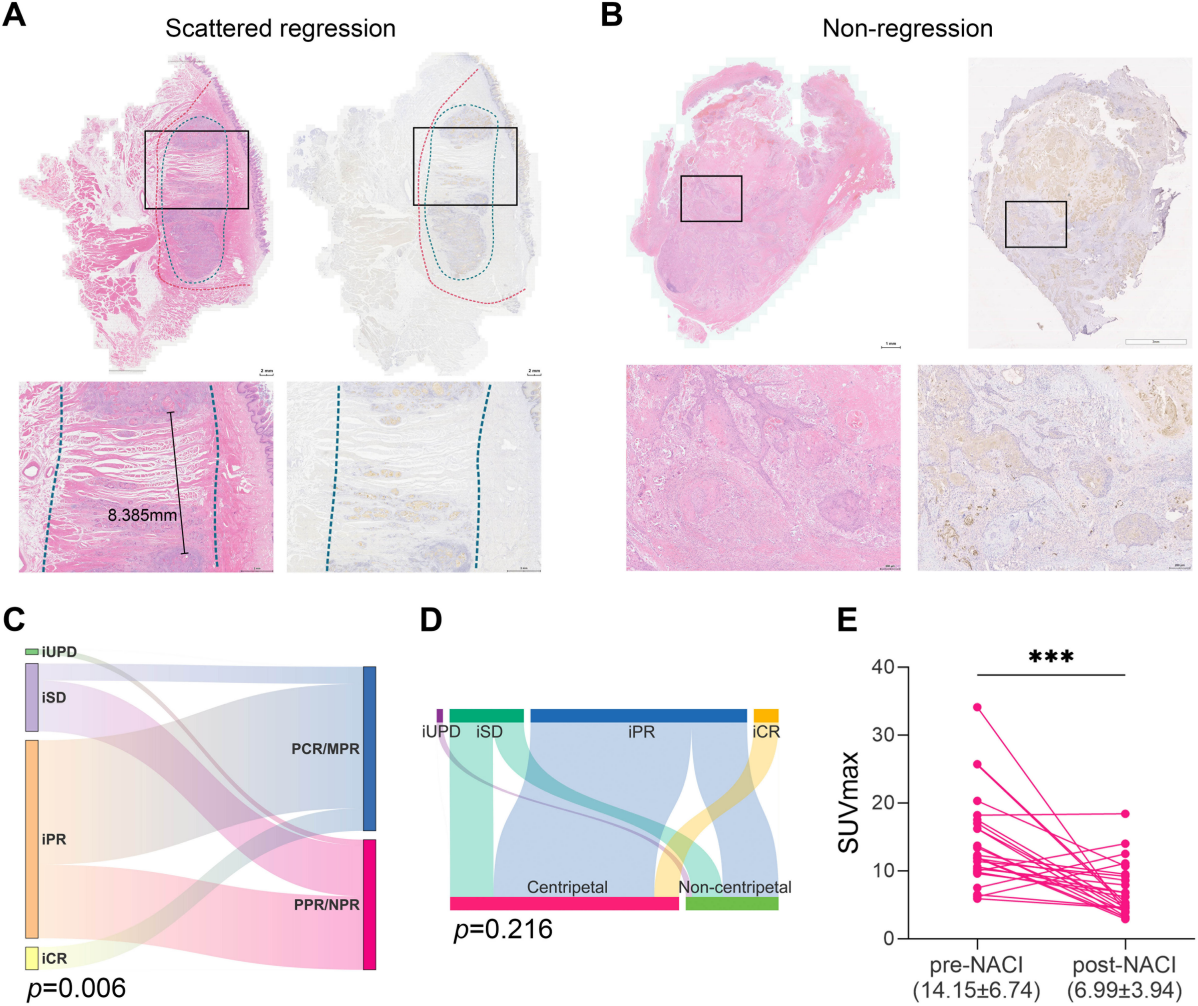
Non-centripetal regression and the correlation between treatment response and regression patterns. Representative image of whole-mount sections showing **(A)** scattered regression (IIa) and **(B)** Non-regression (IIb). Left panel: HE staining, right panel: pan-CK staining. The original tumor bed was outlined by red dotted line. The residual lesion was outlined by blue dotted line. **(C)** Sankey plot showing the correlation between radiological response and pathological response (Chi-square test). **(D)** Sankey plot showing the correlation between radiological response and regression patterns (Chi-square test). **(E)** Comparison of the tumor SUVmax before and after NACI (paired t test, ***, *P*<0.001).

Next, we adopted a whole-mount histopathology approach, which permits comprehensive visualization of the tumor’s histopathological landscape (23), to depict the tumor-regression patterns following NACI. Of these 52 patients, the tumor-regression patterns were classified into two major categories: centripetal regression and non-centripetal regression (**Figure 2**). Centripetal regression was further divided into three subcategories: complete regression (Ia, no viable tumor cells in the whole section, **Figures 2**, **3A**), unifocal centripetal regression (Ib, a unifocal tumor lesion surrounded by a fibrous stroma bed, **Figures 2**, **3B**), and multifocal centripetal regression (Ic, Multiple tumor foci with simultaneous regression were seen in the full field of view, with a fibrous mesenchymal bed visible at the periphery, and the farthest tumor foci were less than 5 mm away from the core tumor foci, **Figures 2**, **3C**). Non-centripetal regression was also divided into two subcategories: scattered regression (IIa, scattered lesions with the distance between the outermost lesion and core lesion is ≥5mm, **Figures 2**, **4A**) and non-regression (IIb, substantial tumor residue was seen in the whole-mount sections, with a rare presence of fibrous stromal bed, **Figures 2**, **4B**). According to this classification, centripetal regression was seen in 37 cases (71.2%), with 8 cases classified as type Ia (15.4%), 19 as type Ib (36.5%), and 10 as type Ic (19.2%) (**Figure 1**). Non-centripetal regression was observed in 15 cases (28.8%), with 13 cases classified as type IIa (25.0%) and 2 cases classified as type IIb (3.8%) (**Figure 1**). Further, we analyzed the overall survival rate and disease-free survival rate between these two groups. The result showed no difference in overall survival and disease-free survival between centripetal regression and non-centripetal regression groups (**Supplementary Figure S2**). Collectively, our data suggests that the centripetal regression represents the predominant tumor regression pattern in post-NACI HNSCC patients.

### 3.4 Potential predictive factors for centripetal regression after NACI

Next, we sought to identify potential predictive factors for centripetal regression after NACI. We first analyzed the correlation between pre-therapeutic clinicopathological parameters and post-NACI tumor regression patterns. As results, patients’ general characteristics such as gender, age, history of tobacco and alcohol consumption, tumor diameter, TNM stage, tumor location, histologic grade, etc., showed no correlation with the tumor regression pattern (**Table 2**). We identified a significant correlation between CPS≥20 and centripetal regression after NACI, as 19 out of 21 cases (90.5%) with CPS≥20 showed a centripetal regression (**Table 2**). We further analyzed the correlation between the tumor regression pattern and post-NACI response. The results revealed a significant association between the centripetal regression pattern and a favorable pathological response to NACI, as 26 out of 27 cases (67.6%) exhibiting centripetal regression achieved PCR or MPR after NACI (**Table 2**).

**Table 2:** Correlation between pre-therapeutic clinicopathological features and post-NACI tumor regression pattern.

Characteristics	Regression pattern			
	N=52	Centripetal (n=37)	Non-centripetal (n=15)	*P* value
Gender				
Male	40	27	13	0.485
Female	12	10	2	
Age				
<60	31	21	10	0.509
≥60	21	16	5	
Tobacco				
No	21	16	5	0.509
Yes	31	21	10	
Alcohol				
No	28	21	7	0.508
Yes	24	16	8	
Areca-nut				
No	41	30	11	0.806
Yes	11	7	4	
HPV status^#^				
Positive	8	3	5	0.085
Negative	34	26	8	
Tumor Diameter				
≤40mm	27	18	9	0.457
>40mm	25	19	6	
T stage*				
T2	1	0	1	0.412
T3	15	11	4	
T4	32	23	9	
N stage				
N0	15	11	4	1.000
N+	37	26	11	
Clinical stage*				
III	12	8	4	1.000
IV	36	26	10	
Histological grade				
Gx/G0	20	14	6	0.885
G1/G2	32	23	9	
Tumor location*				
Tongue	18	12	6	0.362
Buccal	10	7	3	
Gingival	11	9	2	
Oropharyngeal	5	2	3	
Other	4	4	0	
CPS				
<20	31	18	13	**0.011**
≥20	21	19	2	
Radiological response				
iCR/iPR	39	30	9	0.216
iSD/iUPD	13	7	6	
Pathological response				
pCR/MPR	29	25	4	**0.007**
PPR/NPR	23	12	11	

*, 4 patients with metastatic and recurrent tumors were not included in the statistics; #, 10 patients were not applicable of HPV status. Significant P values were marked in bold.

Notably, we observed a correlation between iRECIST-based radiological response and pathological response (**Figure 4C**). Yet the radiological response did not correlate with tumor regression pattern after NACI (**Figure 4D**, **Table 2**). Therefore, we sought to identify an alternative radiologic-based predictive factor for the tumor regression pattern. Analysis of pre- and post-NACI PET-CT revealed a significant decrease in the primary tumor’s maximum standard uptake value (SUVmax), with an average SUVmax of 14.15 ± 6.74 before NACI and 6.99 ± 3.94 after NACI (**Figure 4E**). Furthermore, 10 out of 11 patients with a post-NACI SUVmax reduction rate exceeding 50% demonstrated a centripetal regression pattern following NACI (*P*=0.042, **Table 3**). This correlation suggests that the decrease rate of SUVmax in PET-CT may have significant implications for predicting tumor regression pattern after NACI.

**Table 3:** Correlation between pre-therapeutic clinicopathological features and post-NACI tumor regression pattern.

ΔSUVmax%	Regression pattern			
	N=25	Centripetal (n=17)	Non-centripetal (n=8)	*P* value
≥50%	11	10	1	**0.042**
<50%	14	7	7	

Significant P values were marked in bold.

## 4 Discussion

Previous studies have reported the tumor regression pattern in breast, rectal and esophagus cancers following neoadjuvant chemotherapy, which also mainly include centripetal and non-centripetal regression (17, 18, 20, 21, 24). An early report (25) proposed an MRI-based shrinkage pattern of breast cancer following neoadjuvant chemotherapy: type, I concentric shrinkage without surrounding lesion, type II concentric shrinkage with surrounding lesions, type III shrinkage with residual multinodular lesions, and type IV, diffuse contrast enhancement in whole quadrants. Among them, type IV was more frequently observed in the non-responder group (25). Importantly, radiological scattered regression (type III) might prove to be either complete pathological response (pCR), carcinoma *in situ* (CIS), or residual invasive cancers during histological examination, indicating that common MRI-based evaluation may lead to misjudgment of the pathologic scenarios (25). Therefore, recent studies have focused on the pathological regression pattern. Ling et al. retrospectively analyzed 346 breast cancer patients who received NAC plus breast conservation surgery and classified the regression pattern as pCR, unifocal, limited multifocal and diffuse multifocal regression (21). In esophageal cancer, Tang et al. classified the regression pattern following NAC according to the regression directionality: I, regression toward the lumen; II, regression toward the invasive front; III, concentric regression; IV: scattered regression. And the scattered regression pattern was later showed to associated with increased local recurrence and poor overall survival (18). Additionally, Nagtegaal et al. proposed “mucin pool formation” as an additional post-NAC regression pattern in rectal cancer other than tumor shrinkage (centripetal) and fragmentation (non-centripetal) (24). Here we observed five patterns of post-NACI response: complete regression (pCR), unifocal centripetal regression, multifocal centripetal regression, scattered regression and non-regression. Given that 5mm is a well-recognized safe surgical margin for HNSCC (26, 27), we employed the distance between the outermost lesion and the core lesion exceeding 5mm as a cutoff to discriminate “multifocal centripetal regression” from “scattered regression”. The underlying principle is that although multifocal centripetal regression includes multiple lesions, it still manifests a concentric shrinkage, which potentially permits a reduced surgical margin. Whereas for scattered regression, a reduced resection is precluded as the tumor microfoci might exist beyond the 5mm margin. In addition, previous study demonstrated that the post-NAC diffuse multifocal regression largely associated with ipsilateral breast tumor recurrence after breast-conserving surgery, while limited multifocal regression, unifocal regression and pCR did not (21). This further support the discrimination of “multifocal centripetal regression” from “scattered regression”. Based on the feasibility of a reduced surgical margin, we collectively classified these five patterns into two major categories, the centripetal regression and non-centripetal regression.

Post-NAC non-centripetal regression was reported by previous studies with a variable incidence rate ranging from 18.8% to 71% (16–18, 24, 28, 29), most of them associated with poor prognosis. Notably, a retrospective study showed that multifocal regression was seen in 18.8% of post-NAC HNSCC patients and was an independent predictor for locoregional recurrence (16). This incidence rate is largely proximate to our findings in post-NACI HNSCC patients. Conversely, a recent study in breast cancer showed that non-centripetal regression is more common in post-NACI (68%) patients compared to post-NAC patients (36%) (19). However, of the 23 patients showing post-NACI non-centripetal regression, which was determined by MRI, 19 turned out to reach pCR (19). An earlier study also reported a weak correlation between MRI shrinkage pattern and pathologic response (30). In addition, a low concordance between MRI and pathological shrinkage patterns (48%) after NAC was also reported (25), which again underlines the inconsistency between radiological and pathological evaluation for the regression pattern. In consist with findings in breast cancer, here we observed that the post-NACI regression pattern did not correlate with radiological response, while it significantly associated with pathological response in HNSCC. Such discrepancy highlights that the reliability of MRI-based evaluation of surgical margin is questionable, at least for now.

Hence, an accurate prediction of the pathological regression pattern is essential for precise evaluation of the surgical extent. Former studies have reported that the tumor regression pattern following NAC in breast cancer was correlated with the molecular subtypes, hormone-receptor expression, and MRI pattern enhancement (31–33). Yet the regression pattern discussed in these studies were MRI-based, which largely undermined their value in assessing the surgical margin. Another study in esophageal cancer showed that the fragmented pattern was associated with higher pathological staging and poorer treatment response (17), which consisted with our findings. However, these postoperative pathological indicators had little value in preoperative evaluation. Previous study reported that patients with CPS > 20 in locally advanced HNSCC are more likely to achieve objective response after NACI, though the predictive ability remains weak (34). Importantly, we revealed here that CPS, one of the most crucial indicators for pre-immunotherapeutic evaluation in HNSCC, its score exceeding 20 was significantly correlated with the centripetal regression after NACI, indicating CPS as a potential predictor for post-NACI regression pattern in HNSCC. Nevertheless, this postulation needs to be verified by future studies with a larger sample size and multi-center participation.

We found a significant correlation between the regression pattern and the reduction rate of post-NACI SUVmax in PET-CT. Several studies have reported the predictive value of PET-CT for pathological response after neoadjuvant therapy. In liver-metastatic colorectal cancer patients, those with a SUVmax reduction exceeding 41% after NAC were more likely to achieve pathological response (35). The parameters of PET-CT demonstrate excellent performance in predicting pCR after NACI in esophageal squamous cell carcinoma, post therapeutic SUV_max_, SUV_mean_, SUV_TBR_, TLG, and MTV showed excellent predictive value for the pCR of primary tumors (36). Additionally, in non-small cell lung cancer (NSCLC), the efficacy evaluation of PET/CT based on PERCIST criteria is highly consistent with postoperative pathological following NACI, and a ΔSUV-based predictive model for postoperative pathological response showed high sensitivity and specificity (37). Notably, only a few cases showed consistency between iRECIST-based radiological response and PERCIST-based PET-CT evaluation as well as pathological response. Cheng et al. proposed that one possible reason for this difference is that PET reflects the biological activity of the tumor, hence PET/CT may be a more accurate reflection of residual viable tumor cells comparing to CT, which mainly reflects the anatomic properties of lesion (37). This may also explain the higher SUVmax reduction in centripetally regressed tumors, as they tend to have less residual viable tumor cells compared to non-centripetally regressed ones. Our data indicated that PET-CT might be a feasible approach for predicting tumor regression pattern and further assist the surgical decision.

## 5 Conclusions

This pilot study described the pathological tumor regression patterns as well as the ratio of each pattern following NACI. Centripetal regression was identified as the dominant pattern of regression after NACI, thus presumably supporting a reduced surgical margin in these patients. However, for those patients who may present with non-centripetal regression, expanded resection of the original extent as well as post-surgical radiotherapy, immunochemotherapy, and targeted therapy may be of importance. Additionally, we identified that pre-therapeutic CPS and the reduction rate of post-NACI SUVmax are potential predictive factors for post-NACI centripetal regression in HNSCC. Our study provides preliminary evidence for reduced surgical extent in post-NACI HNSCC patients. Future studies with larger sample size and multi-center involvement are required to uncover accurate predictive factors for the regression pattern, which may eventually assist in risk stratification and surgical decision making in post-NACI HNSCC patients.

## Data Availability

The original contributions presented in the study are included in the article/**Supplementary Material**. Further inquiries can be directed to the corresponding authors.

## Supplementary material

Supplementary Figure 1 The process of whole-mount histopathology.

Supplementary Figure 2 Analysis of the overall survival and disease-free survival between centripetal regression and non-centripetal regression groups.
