# Supplementary material for: Pathological regression patterns following neoadjuvant chemo-immunotherapy in head and neck squamous cell carcinoma: a pilot study
Source: Front Immunol. 2025 Aug 6;16:1627442. doi: 10.3389/fimmu.2025.1627442 (PMC12364697; doi:10.3389/fimmu.2025.1627442)
Supplement: Supplementary file 1 [file Table1.pdf]

Table S1. Treatment-related adverse events (TRAEs) during neoadjuvant therapy

TRAEs, n (%)	Any grade	Grade1-2	Grade 3	Grade 4
Alopecia	52 (100)	52(100)	0	0
Fatigue	24 (46.2)	24 (46.2)	0	0
Rash	24 (46.2)	21 (40.4)	3(5.8)	0
Hepatic dysfunction	22 (42.3)	17 (32.7)	5(9.6)	0
Anorexia	19 (36.5)	19 (36.5)	0	0
Fever	10 (19.2)	8 (15.4)	0	0
Myelosuppression	8 (15.4)	5 (9.6)	0	2 (3.8)
Nausea/vomiting	7 (13.5)	7 (13.5)	0	0
Arthralgia	7 (13.5)	7 (13.5)	0	0
Myalgia	5 (9.6)	5 (9.6)	0	0
Diarrhea	5 (9.6)	5 (9.6)	0	0
Cardiotoxicity	5 (9.6)	3 (5.8)	1 (1.9)	0
Renal dysfunction	3 (5.8)	3 (5.8)	0	0
Constipation	3 (5.8)	3 (5.8)	0	0
Hypothyroidism	4 (7.7)	3 (5.8)	0	0
Adrenocortical hypofunction	3 (5.8)	2 (3.8)	0	0
Xerophthalmia	1 (1.9)	1 (1.9)	0	0
Xerostomia	1 (1.9)	1 (1.9)	0	0
