# Supplementary figures and images for: Pathological regression patterns following neoadjuvant chemo-immunotherapy in head and neck squamous cell carcinoma: a pilot study
Source: Front Immunol. 2025 Aug 6;16:1627442. doi: 10.3389/fimmu.2025.1627442 (PMC12364697; doi:10.3389/fimmu.2025.1627442)

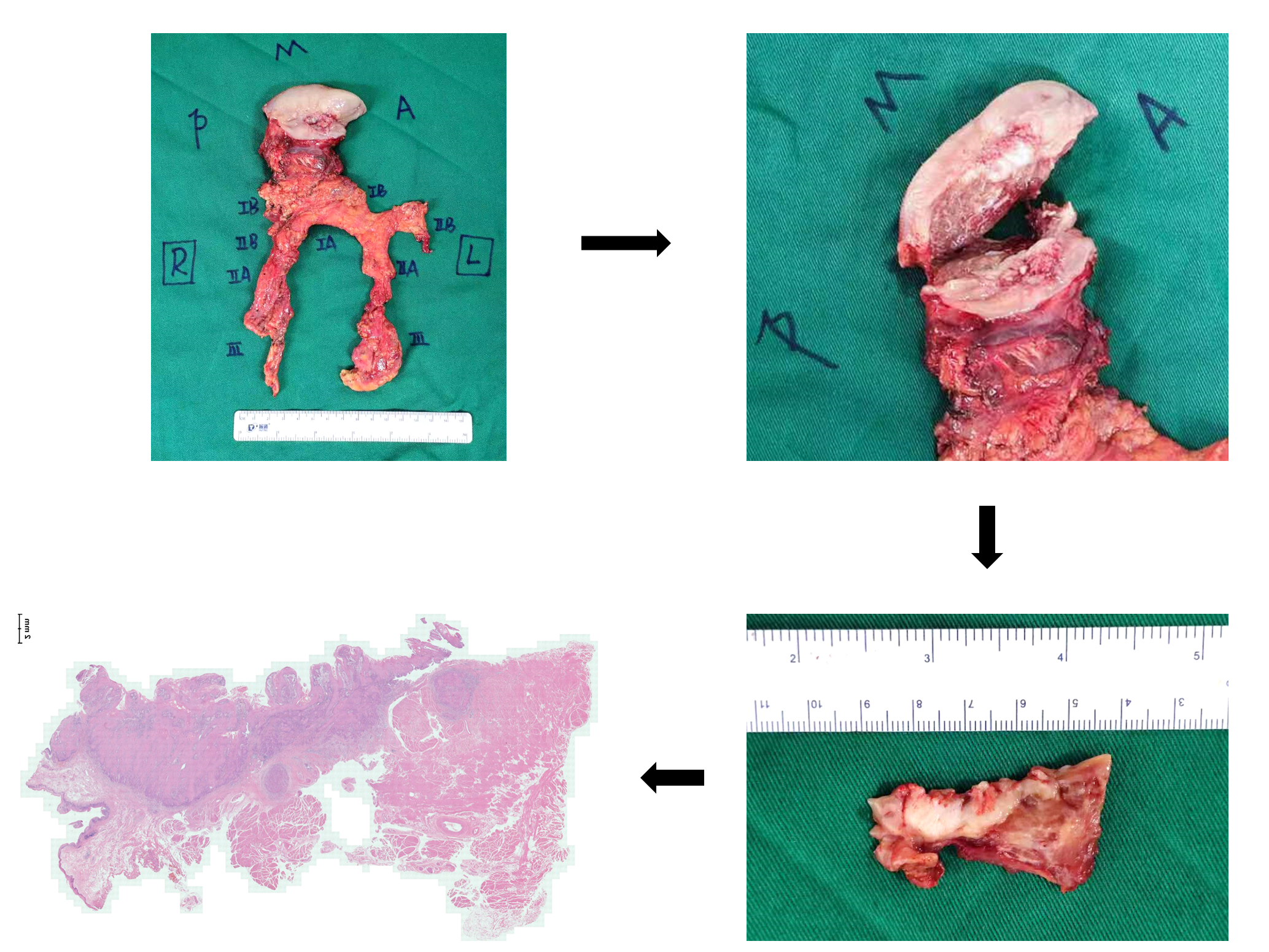

Supplement: Supplementary Figure 1 — The process of whole-mount histopathology. [file Image1.tif]

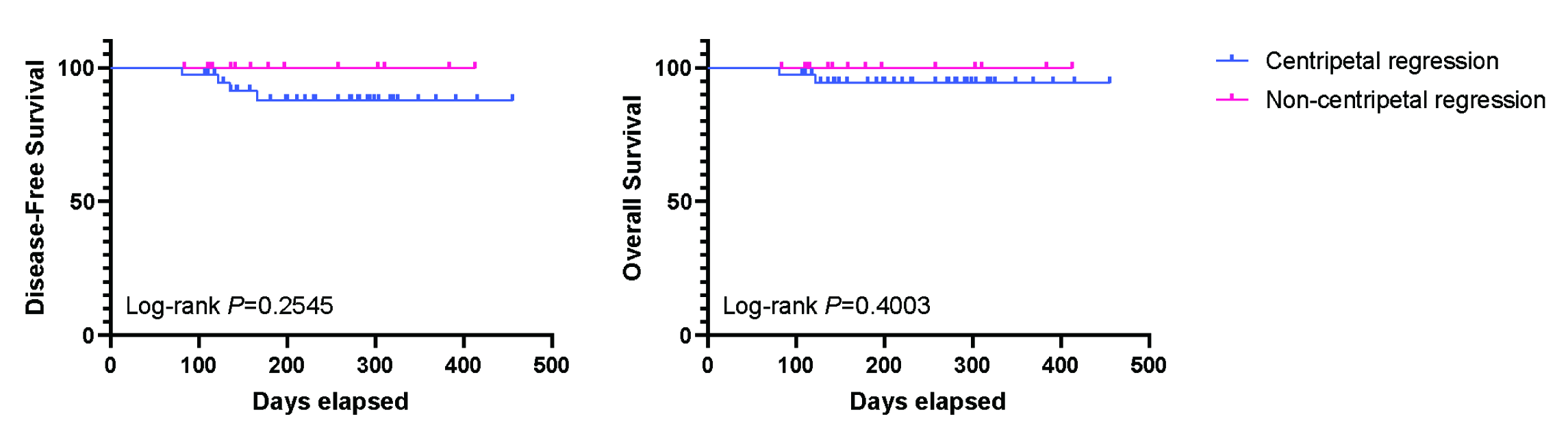

Supplement: Supplementary Figure 2 — Analysis of the overall survival and disease-free survival between centripetal regression and non-centripetal regression groups. [file Image2.tif]
